# Hidden Fungal DNA Structures May Shape Sequencing Outcomes

**DOI:** 10.1002/bies.70153

**Published:** 2026-06-15

**Authors:** Paul W. Thomas

**Affiliations:** ^1^ Faculty of Natural Sciences University of Stirling Stirling UK; ^2^ Mycorrhizal Systems Ltd Lancashire UK

**Keywords:** DNA, fungi, nanopore, novel structures, sequence artefacts

## Abstract

Fungal DNA is systematically under‐detected in shotgun metagenomics, likely due in part to physical barriers like melanized cell walls and complex DNA conformations. Additionally, Oxford Nanopore Technologies sequencing with native fungal DNA often results in rapid pore clogging and unusual translocation dynamics, possibly due to intrinsic, yet undescribed, structural complexities. Exploring these signals could reveal novel fungal genome architectures, enhance sequencing accuracy, and drive advances in fungal biology.

## Graphical abstract

Sequencing fungal DNA presents numerous challenges, and unexplored structural anomalies may be a contributor. Native ONT sequencing allows artefacts of disrupted pore translocation to be interrogated, facilitating mapping to genomic loci using raw signal analyses. Subsequent visualization tools could reveal novel genome architectures and drive advances in fungal biology.

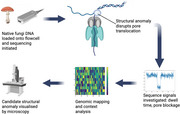

## 1 Main Text

Fungal biomass is ubiquitous, from forest soils to plant roots, and often visibly dominant in environmental samples. Yet shotgun metagenomic sequencing routinely yields unexpectedly few fungal reads, even in substrates visibly replete with mycelium [1, 2]. PCR‐based amplification of the ITS region can increase apparent detection, but it introduces well‐known artefacts: primer bias, taxonomic blind spots, chimeras, shortened reads, and loss of base modifications such as methylation [3]. Without amplification, fungi appear to be systemically under‐detected, and this discrepancy may hint at structural, not solely technical, biases.

Part of the challenge lies in physical barriers. Fungal cells are armored with chitinous, glucan‐rich, and often melanized walls, and they are frequently embedded in complex soil matrices, shielding nuclei from lysis [4]. For these reasons, targeted protocols often depend on mechanical disruption, such as bead‐beating, for effective fungal DNA recovery [5]. Beyond cellular protection, fungal DNA itself carries intrinsic complexities. DNA methylation varies widely across fungal taxa but is often concentrated on transposons and repetitive regions [6], and repetitive, or GC‐rich regions may form hairpins, G‐quadruplexes, or other non‐B conformations [7]. Multinucleate fungi and intron‐rich mitochondrial genomes further complicate genomic representation [8]. Collectively, these features may influence how fungal DNA interacts with purification columns and sequencing platforms, biasing recovery and representation.

Observations from colleagues and my own laboratory using Oxford Nanopore Technologies (ONT) sequencing suggest a further anomaly: native fungal DNA frequently causes unexpectedly rapid flow‐cell degradation, pore clogging, and low reuse, in stark contrast to amplified, bacterial, or plant DNA. Part of the issue may lie in contamination carried through from the DNA extraction process itself, and mitigation measures have been proposed and tested [5]. However, these phenomena may not be mere technical annoyances; they could also reflect fundamental physical differences in fungal DNA behavior through nanopores. Nanopore sequencing works by ratcheting DNA through a nanoscale pore while detecting base‐specific ionic current disruptions [9]. Translocation dynamics—dwell time, event duration, and pore blockage—capture information not just on nucleotide sequence but also on physical attributes of the molecule [9]. Emerging research indicates that non‐B DNA structures, such as hairpins and G‐quadruplexes, can measurably alter nanopore translocation times [7]. Thus, ONT data inherently encode structural signatures that remain invisible in conventional downstream analysis. Further, it is worth noting that such contrasts between the sequencing of native and amplified DNA are not solely an ONT issue; they are also noted in long‐read technology by Pacific Biosciences. In this case, an amplification‐based protocol has been presented as a solution, but this approach again removes biologically relevant structural and mechanical anomalies, such as base modification [10]. Nevertheless, the cross‐platform occurrence of these sequencing contrasts supports the case for further causal investigation.

**FIGURE 1:**
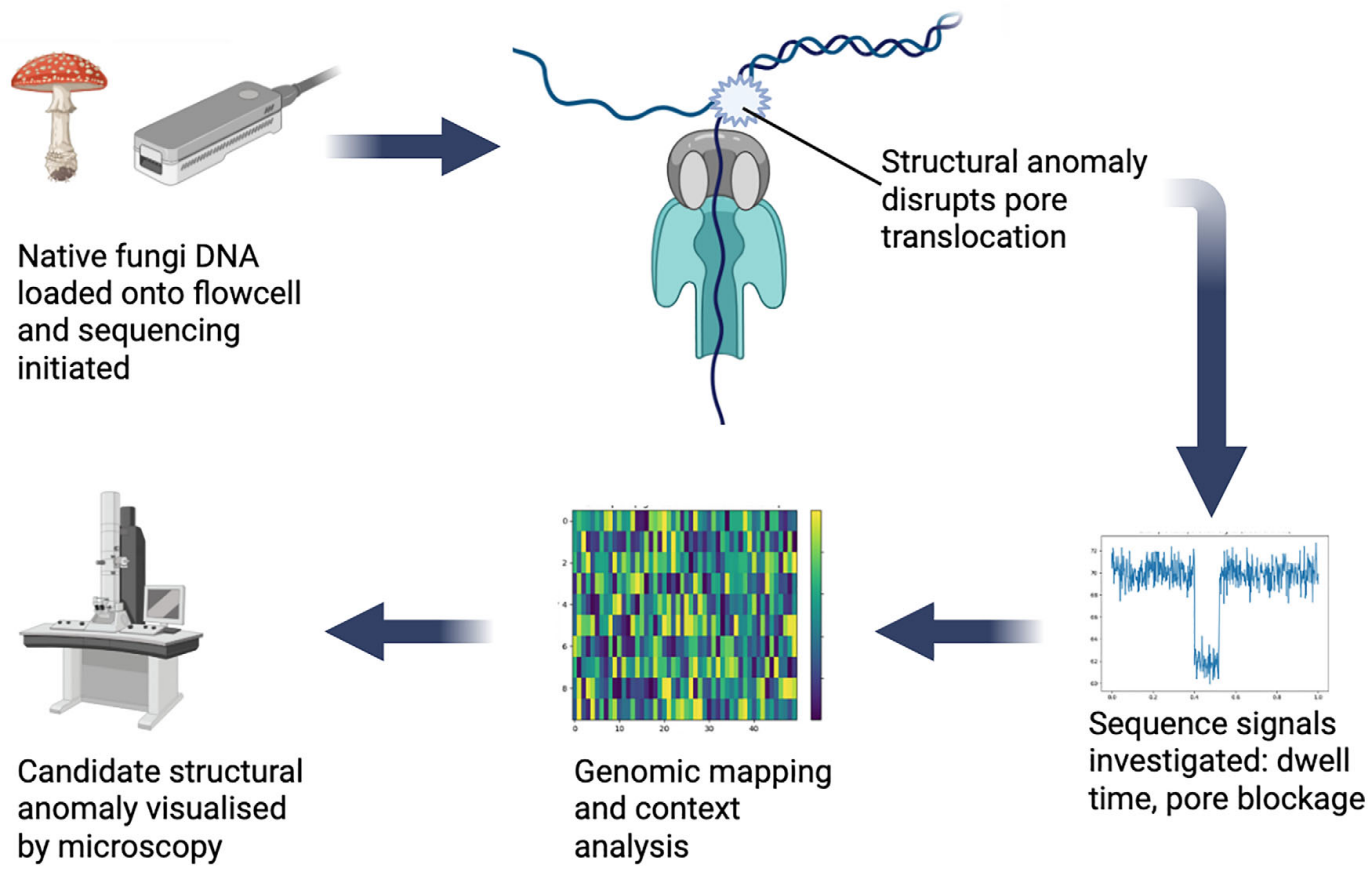
Conceptual framework for detecting structural features in fungal DNA using nanopore sequencing. Intrinsic structural features may disrupt pore translocation, producing characteristic signal‐level artefacts such as prolonged dwell times or pore blockages. These anomalies can be mapped to genomic loci using raw signal analyses and visualisation tools, enabling prioritisation of candidate regions for orthogonal structural characterisation using microscopy‐based approaches.

These insights present key opportunities. Event‐level information preserved in native‐DNA ONT datasets can be interrogated with established pipelines such as Nanopolish eventalign [11] or the fast5_research API [12], enabling direct extraction of dwell‐time and event‐duration statistics. Visual tools such as BulkVis further allow localization of problematic regions by mapping raw signal traces to genomic coordinates [13]. Together, these analyses could move beyond anecdotal observations of pore failure to systematically map the structural fingerprints of fungal DNA at identified causal loci and uncover architectural features that currently remain invisible in conventional sequencing (Figure 1). Even existing, unamplified ONT datasets could yield preliminary insights into fungal structural dynamics with minimal additional laboratory effort.

Moving forward, experimental benchmarking should systematically compare identical genomic loci across multiple fungal genets to determine whether anomalous translocation metrics are reproducibly associated with specific sequence contexts or chromosomal regions. Systematic locus‐level comparisons utilizing PCR‐free, long‐read nanopore libraries with targeted enrichment (e.g., adaptive sampling) or careful mapping of whole‐genome reads—will reveal whether dwell‐time deviations, recurrent current disruptions, or pore blockages co‐localize to the same coordinates within and between genets. Once candidate loci are identified from raw “squiggle” analyses, inspection with BulkVis and aggregation of per‐event statistics can prioritize sites for structural characterization. This aggregation step would require the development of a bespoke script, but this is a relatively simple process given the open nature of BUlkVis and the use of the Python 3 programming language [14]. Direct single‐molecule visualization, such as atomic force microscopy or electron microscopy, can then validate the inferred structures at those precise loci [13, 15]. Demonstrating locus‐specific, conserved translocation anomalies would (1) strengthen the case that fungal genomes harbor recurrent structural features that perturb nanopore sequencing, (2) enable targeted protocol adjustments to mitigate flow‐cell damage, and (3) provide a tractable route to discover novel DNA conformations with the potential to significantly advance knowledge of genome regulation and architecture.

Fungi have long been understudied in structural genomics relative to bacteria and mammals. The idea that the DNA structure itself, not merely quantity, drives under‐detection in sequencing is novel and requires attention. By reinterpreting nanopore sequencing artefacts as structural signals, researchers could simultaneously address a persistent technical blind spot and uncover fundamental aspects of fungal genome biology. Unlocking these structural characteristics has the potential to transform mycological research, bridging technical challenges with new perspectives on fungal genome biology. At the very least, understanding the structural causes of poor fungal recovery may inform optimized extraction protocols or pore chemistries, while more accurate fungal sequencing would benefit pathogen surveillance, ecological monitoring, and biotechnology applications.

## Author Contributions

**Paul W. Thomas**: conceptualised, wrote and edited the article as sole author.

## Conflicts of Interest

The author declares no conflicts of interest.

## Data Availability

Data sharing is not applicable to this article as no datasets were generated or analyzed during the current study.
